# Comment on Lau et al. Trends in Beef Intake in the United States: Analysis of the National Health and Nutrition Examination Survey, 2001–2018. *Nutrients* 2023, *15*, 2475

**DOI:** 10.3390/nu15183935

**Published:** 2023-09-11

**Authors:** Katherine Consavage Stanley, Vivica I. Kraak

**Affiliations:** Department of Human Nutrition, Foods, and Exercise, Virginia Polytechnic Institute and State University (Virginia Tech), Blacksburg, VA 24061, USA; vivica51@vt.edu

We were interested to read the results of Lau et al., 2023 [1] on United States (U.S.) beef intake trends (2011–2018). However, we were surprised that these investigators did not mention the environmental effects of producing and consuming beef, which requires substantial land and water use and contributes to global greenhouse gas emissions, eutrophication, and biodiversity loss [2,3]. Consensus recommendations among expert bodies clearly encourage populations to reduce red meat intake to support human and planetary health goals [4,5,6]. While the U.S. Dietary Guidelines for Americans (DGA) 2020–2025 report did not mention the environmental impact of dietary patterns [7], climate and public health groups have called for the U.S. government to incorporate sustainability principles into national dietary guidance, as many other high-income countries have carried out [8], and to recommend a shift from red meat to plant-based proteins [9].

The U.S. beef industry has used the Cattlemen’s Beef Board to downplay beef’s sustainability impacts [10]. The Lau et al. study was funded by the Beef Checkoff Program [1]. The Checkoff Program oversees U.S. beef marketing and promotion efforts that aim to increase U.S. and international beef consumption, including the “Beef—It’s What’s for Dinner” campaign that has run for three decades [11]. The Beef Checkoff Program reported 2022 revenue of USD 43.8 million, with spending overseen by the Cattlemen’s Beef Board and the U.S. Department of Agriculture, the latter of which also oversees the DGA process [12].

Lau et al., 2023 [1] claimed that “beef is not overconsumed” in the U.S. This is based on the finding that 31% of beef-consuming adults’ (i.e., those aged 19–59 years old) and 19% of older adults’ (i.e., those ≥60 years old) daily beef intake met or exceeded the 3.7 ounces/day of collective lean meat, poultry, and/or egg intake modeled in the DGA’s Healthy U.S.-Style Dietary Pattern (HDP). Yet this assumes no daily intake of other red meat, poultry, or eggs. When compared to the HDP’s 1.8 ounce/day allotment for lean red meat, Lau et al. found that 95% of beef-consuming adults and 94% of older adults exceeded this level [1]. Current U.S. beef intake levels also far exceed the EAT-Lancet Commission’s planetary health diet guidelines, which encourage no more than 14–28 g (0.5–1 ounce) of red meat intake daily [4,8]. The reduction in beef intake identified from 2001–2018, although statistically significant, equated to a 0.2-ounce decrease among adults and no change among older adults [1]. When contextualized within the current global discourse to transform food systems under a changing climate, Lau et al.’s findings suggest that the U.S. government and businesses must accelerate efforts to enable Americans to change their beef purchase and consumption habits to support the long-term health of people and the planet.
